# Spatially‐Resolved Organoid Transfection by Porous Silicon‐Mediated Optoporation

**DOI:** 10.1002/adma.202407650

**Published:** 2024-10-17

**Authors:** Chantelle Spiteri, Valeria Caprettini, Yikai Wang, Sofia Dominguez‐Gil, Martti Kaasalainen, Cong Wang, Davide Alessandro Martella, Samuel McLennan, Priya Vashisth, Magali Gary‐Bobo, Christophe Nguyen, Mads Bergholt, Jean‐Olivier Durand, Frédérique Cunin, Ciro Chiappini

**Affiliations:** ^1^ Centre for Craniofacial and Regenerative Biology King's College London London SE1 9RT United Kingdom; ^2^ London Centre for Nanotechnology King's College London London WC2R 2LS United Kingdom; ^3^ ICGM Univ. Montpellier CNRS ENSCM Montpellier 34293 France; ^4^ IBMM Univ. Montpellier CNRS ENSCM Montpellier 34293 France

**Keywords:** gene delivery, optoporation, organoids, porous silicon, transfection

## Abstract

Engineering the spatial organisation of organotypic cultures is pivotal for refining tissue models that are useful for gaining deeper insights into complex, non‐cell autonomous processes. These advanced models are key to improving the understanding of fundamental biological mechanisms and therapeutic strategies. Controlling gene regulation through spatially‐resolved delivery of nucleic acids provides an attractive approach to produce such tissue models. An emerging strategy for spatially‐resolved transfection uses photosensitizing nanoparticles coupled with laser pulses to optoporate cells in culture and locally mediate gene delivery. However, localized optoporation in 3D systems remains challenging. Here we propose a solution to this longstanding hurdle, demonstrating that porous silicon nanoparticles are a safe and bioresorbable photosensitising nanomaterial capable of spatially‐resolved transfection of mRNA in MCF‐7 organoids by near‐infrared two‐photon optoporation. Functionalization with an azobenzene–lysine photo‐switchable moiety enhances the transfection efficiency of the nanoparticles up to 84% in a 2D cell system. Moreover, the nanoparticles enable spatially selective mRNA transfection to MCF‐7 spheroids, demonstrating targeted  gene delivery in complex 3D cellular environments. The approach for spatially‐resolved 3D optoporation offers a way forward for the design of tailored spheroids and organoids by spatially selective nucleic acids delivery.

## Graphical abstract

Precise control over gene delivery in 3D can refine tissue models providing insights into the complex, multicellular processes underlying development, degeneration, and disease progression. This work presents an optoporation strategy based on light‐responsive porous silicon nanoparticles for the spatially‐resolved transfection of organoids.

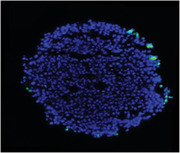

## 1 Introduction

Modeling the structure and function of biological systems requires recapitulating precise spatiotemporal coordination across many different cells.^[^ ^1^ ^]^ For example, orchestrating development relies on localized signaling centers that supply morphogen gradients to guide structural patterning during embryogenesis in a tightly regulated fashion.^[^ ^2^, ^3^ ^]^ In cancer, the complex tumor microenvironment and the intricate tumor‐stromal interactions involving cancer‐associated fibroblasts, immune cells, and endothelial cells drive tumor progression, invasion, and metastasis.^[^ ^4^ ^]^ Developing this ability to precisely manipulate cellular functions in complex systems could provide critical insights into physiological and disease mechanisms^[^ ^5^ ^]^ leading to progress in advanced therapeutics and fundamental biology research.^[^ ^6^, ^7^ ^]^

The growing interest in studying these complex, multi‐cellular events has been enabled by the rapidly advancing ability to generate organotypic models in vitro.^[^ ^8^ ^]^ However, the representativeness of these models isreduced by our limited ability to coordinate their component cells. A plethora of bioengineering and cell biology approaches have been developed to improve organotypic modeling, however a precise control over cell fate within these systems remains elusive. Gaining precise control over single‐cell gene expression in time and space could provide a transformative advance in regulating cell fate and enhancing organotypic in vitro models for studying complex multicellular processes.^[^ ^9^ ^]^ Spatially‐resolved nucleic acid delivery could achieve this goal. However, many of the leading approaches for nucleic acid delivery, including viral and non‐viral vectors, electro‐ and sono‐poration, have limited spatial selectivity, having been developed for bulk, high‐throughput transfection.^[^ ^10^, ^11^ ^]^ Approaches for single cell‐transfection such as micropipettes, FluidFM, and the Scanning ion Conductance Microscope are poorly amenable to 3D systems.^[^ ^12^, ^13^ ^]^ As a result, achieving high‐efficiency intracellular delivery with spatiotemporal precision at the single‐cell level in organoids remains challenging. Indeed, there is often a trade‐off between high‐throughput bulk techniques lacking control, versus low‐throughput precise single‐cell delivery.^[^ ^14^, ^15^ ^]^

Cellular engineering approaches are emerging to tackle this issue. Optogenetics combined with CRISPR enables spatially‐resolved, light‐inducible activation and repression of target genes.^[^ ^16^, ^17^ ^]^ However, such an approach requires genetic engineering of cells, which is cumbersome and not universally accessible, particularly for the most relevant, human‐ and primary cell‐derived systems. In addition, blue light activation is required which is heavily absorbed by the cell resulting in increased phototoxicity.^[^ ^18^ ^]^

Optoporation holds the potential to develop a universal approach for 3D spatiotemporal control of gene expression. Optoporation is a non‐contact approach that can transfect a variety of nucleic acids including siRNA,^[^ ^19^, ^20^, ^21^ ^]^ mRNA,^[^ ^22^, ^23^, ^24^, ^25^ ^]^ and plasmids^[^ ^26^, ^27^, ^28^, ^30^, ^31^, ^39^ ^]^ in a wide range of cells with single‐cell resolution and temporal control. The throughput and efficiency of optoporation are greatly improved by the use of sensitizing nanomaterials, the most common being gold and carbon‐based nanoparticles. However, these nanomaterials are often not biodegradable and can be genotoxic^[^ ^32^, ^33^, ^34^ ^]^ limiting their applicability for advanced therapies and accurate modeling.

Ultimately, the spatially‐resolved optoporation of 3D organotypic systems remains elusive. Porous silicon nanoparticles are a promising nanomaterial for sensitized optoporation as they are bioresorbable^[^ ^35^, ^36^ ^]^ and can be excited by near‐infrared two‐photon irradiation^[^ ^37^ ^]^ to generate reactive oxygen species and localized heating.^[^ ^38^ ^]^ The coupling of photosensitisers such as porphyrin rings further enhances the ability of porous silicon to generate reactive oxygen species.^[^ ^39^, ^40^, ^41^, ^42^ ^]^ This absorption of near‐infrared two‐photon light can be leveraged to promote nucleic acid delivery in cancer cells.^[^ ^43^ ^]^ Additionally, porous silicon nanoparticles possess a tuneable large surface area and pore volume^[^ ^44^ ^]^ providing capacity for loading nucleic acids and controlling their delivery,^[^ ^45^, ^46^, ^47^, ^48^, ^49^, ^50^, ^51^ ^]^ alongside a versatility for surface functionalisation.^[^ ^52^, ^53^ ^]^ This approach using porous silicon nanoparticles for optoporation‐mediated gene delivery holds promise for refining in vitro and ex vivo models. In developmental biology, it could control morphogen expression to regulate embryo polarisation. In disease modeling, it could localize the onset of mutations that drive cancer progression and metastasis, or guide the polarisation of immune cells to investigate their role in immunotherapies. The system's non‐viral, biodegradable nature provides a safer alternative to traditional methods, enhancing its potential for clinical translation.

Here, we report the use of porous silicon nanoparticles as optoporation sensitizers enabling high‐efficiency, spatially‐resolved transfection in 2D and 3D cellular systems. We systematically optimised optoporation parameters including laser power and scanning patterns, nanoparticle concentration, and surface functionalization. We generated 3‐aminopropyltriethoxysilane (APTES) and light‐responsive isocyanopropyltriethyoxysilane–azobenzene–lysine (AzoLys) functionalized nanoparticles to evaluate the role of mRNA complexation and light‐triggered mRNA release on transfection efficiency. We established the conditions to efficiently mediate transfection without generating reactive oxygen species and inducing apoptosis. In these conditions, the AzoLys nanoparticles outperformed APTES nanoparticles achieving GFP‐mRNA transfection efficiency of up to 84% in MCF‐7 breast cancer cells. Guided by this information, we transfected selected cells within a 3D spheroid of MCF‐7 breast cancer cells showcasing the potential for targeted gene delivery in the tumour microenvironment. Altogether, this study identified a biodegradable nanomaterial capable of mediating optoporation, resulting in high mRNA transfection efficiency and the spatially‐resolved transfection of 3D cellular systems.

## 2 Results

### 2.1 Fabrication and Characterization of Porous Silicon Nanoparticles

**Figure 1:**
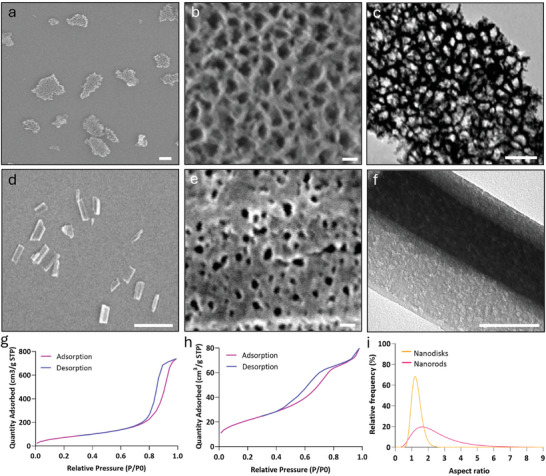
Characterization of porous silicon nanoparticles for optoporation. Characterization of a,b,c) nanodisks and d,e,f) nanorods by a,b,d,e) scanning electron microscopy (SEM), c,f) transmission electron microscopy (TEM), and g,h) nitrogen sorption isotherm. i) Comparison of nanodisks and nanorods aspect ratio calculated from SEM analysis. Data was collected from the measurement of 450 nanoparticles for each nanodisks and nanorods. Scale bars: a) 200 nm, b,e) 20 nm, c,f) 100 nm, d) 1 µm.

We fabricated porous silicon nanoparticles of different geometries to explore the role of shape in permeabilising cells when irradiated with a femtosecond laser. Specifically, we produced discoidal‐like (nanodisks) and rod‐like (nanorods) nanoparticles. Nanodisks (**Figure** **1**a) were fabricated by electrochemical etching to produce nanoparticles with an average hydrodynamic diameter of 282 ± 2.5 nm, a specific surface area of 344.5 m^2^ g, and a PDI of 0.2 ± 0.02. Additionally, nitrogen sorption isotherms indicated 65% porosity and ≈12 nm average pore diameter, aligning with the pore sizes observed by scanning electron microscopy (SEM) and transmission electron microscopy (TEM) (Figure 1b,c,g). The nanorods (Figure 1d) were fabricated through metal‐assisted chemical etching. Per SEM analysis, these measured an average length and width of 404 and 171 nm respectively. In this case, nitrogen sorption isotherms indicated a porosity of 50%, a specific surface area of 77.2 m^2^ g^−1^ and an average pore diameter of ≈7.9 nm which aligned with the pore size observed by SEM and TEM (Figure 1e,f,h). Nanodisks had a 1.3 ± 0.3 aspect ratio compared to a 2.2 ± 1.4 aspect ratio for nanorods (Figure 1i). To promote their interaction with nucleic acids and the cell membrane, nanoparticles were functionalized with APTES acquiring a surface charge of 27.3 mV ± 0.6 for nanodisks and 21.3 mV ± 1.0 for nanorods.

The selection of nanoparticle parameters in our study was guided by multiple factors aimed at optimizing optoporation. We targeted a pore size range of 8–12 nm, which has been shown to effectively generate singlet oxygen and disturb cell membranes.^[^ ^54^ ^]^ The 200–300 nm size reduced uptake kinetics while maintaining good cytocompatibility. While 50 nm nanoparticles exhibit optimal cellular uptake,^[^ ^55^ ^]^ increasing size to 300 nm reduces uptake to a minimum.^[^ ^56^, ^57^ ^]^

### 2.2 Cell‐Nanoparticle Interaction

**Figure 2:**
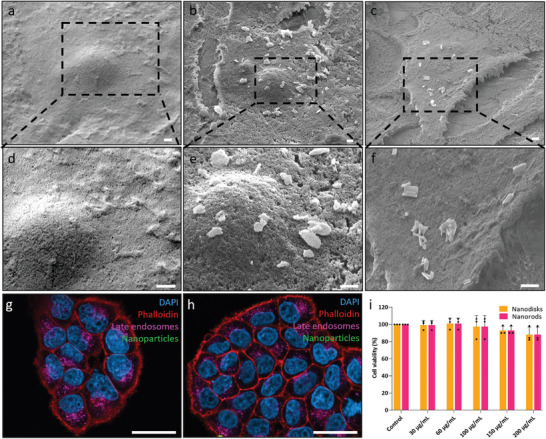
SEM images of the MCF‐7 with and without nanoparticles. a,d) SEM imaging of MCF‐7 cells without nanoparticles, b,e) MCF‐7 cells after 1 h incubation with 30 µg mL^−1^ of nanodisks. c,f) MCF‐7 cells after 1 h incubation with 30 µg mL^−1^ of nanorods. Confocal imaging of MCF‐7 cells g) without nanoparticles and h) with nanoparticles after 1 h incubation with nanodisks. Scale bars: a–f) 1 µm, g,h) 50 µm. i) Cell viability assay examining cytotoxicity of nanoparticles in MCF‐7 cells as a function of concentration. Viability is expressed relative to an untreated control. Data was collected from N = 3 biological replicates. Statistical analysis by ordinary two‐way ANOVA with Tukey's post‐hoc test was performed to determine significant differences, data shown as mean with ± standard deviation.

For efficient optoporation, it is important that nanoparticles adhere to the membrane of healthy cells. SEM imaging confirmed the adherence of nanoparticles to the MCF‐7 cell membrane after 1 h of incubation (**Figure** **2**a–f), with energy dispersive X‐ray (EDX) analysis corroborating their silicon composition (Figure S1, Supporting Information). After 24 h, SEM imaging showed a significant reduction in the membrane‐bound nanoparticles, with only a few remaining on the cell surface (Figure S2a,b, Supporting Information). This observation was consistent with confocal microscopy images, which showed a substantial increase in internalised FITC‐tagged nanoparticles between 1 h (Figure 2g,h) and 24 h (Figure S2c,d, Supporting Information). We also assessed the potential cytotoxicity by measuring MCF‐7 viability following a 24 h incubation with the nanoparticles. Cell viability remained comparable to untreated controls up to a nanoparticle concentration of 200 µg mL^−1^ for nanodisks and nanorods alike (Figure 2i).

### 2.3 Optimisation of Optoporation Parameters

**Figure 3:**
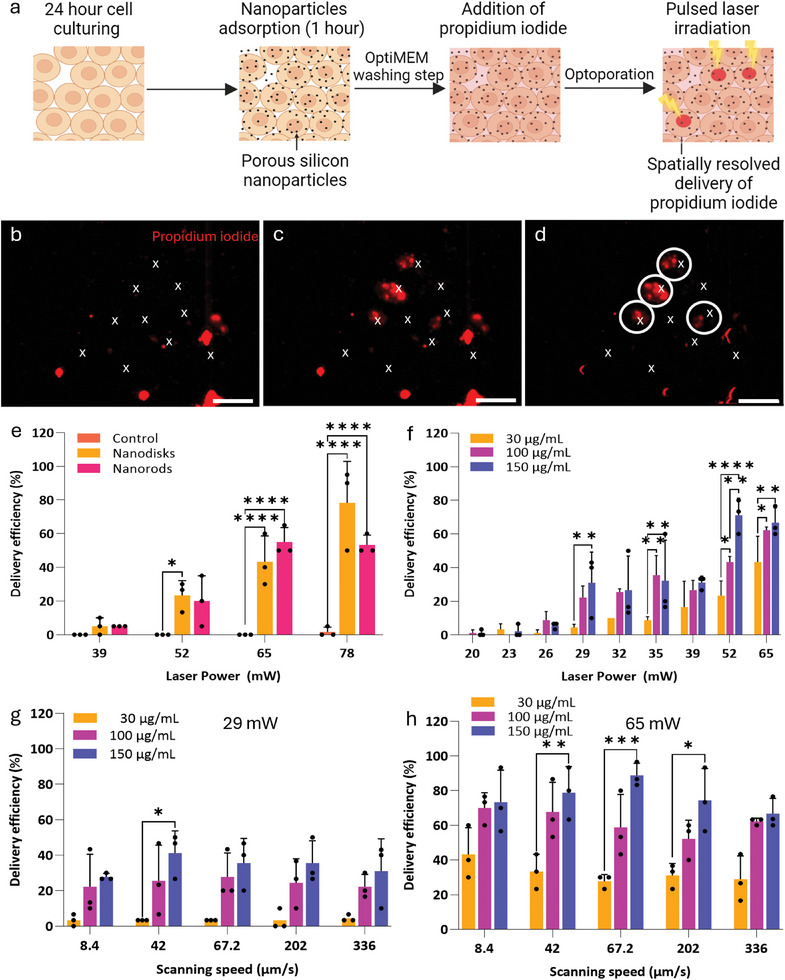
Optimization of optoporation conditions. a) Schematic illustration of the optoporation procedure for propidium iodide delivery. Created with BioRender.com. b–d) Fluorescence microscopy images showing propidium iodide uptake by cells b) prior to optoporation to exclude dead cells, c) following optoporation showing propidium iodide delivery, d) subtraction of pre‐optoporation from post‐optoporation to highlight optoporated cells. White x marks indicate optoporation targets; white circles highlight optoporated cells. Scale bar: 100 µm. e) Quantification of delivery efficiency as a function of laser power in the presence of 30 µg mL^−1^ of nanoparticles. Control: no nanoparticles. f) Quantification of delivery efficiency as a function of laser power and nanoparticle concentration. g,h) Quantification of delivery efficiency as a function of laser scanning speed and nanoparticle concentration at g) 29 and h) 65 mW. e–h) Data was collected from *N* = 3 biological replicates. Statistical analysis by ordinary two‐way ANOVA with Tukey's post‐hoc test was performed to determine significant differences (*p < 0.05, **p < 0.01, ***p < 0.001, ****p < 0.0001). Data shown as mean with ± standard deviation.

To evaluate the feasibility of optoporation using porous silicon nanoparticles, we assessed the delivery of propidium iodide to MCF‐7 adherent breast cancer cells. The cells were incubated with 30 µg mL^−1^ of nanoparticles for 1 h. After washing away unbound nanoparticles, each cell was optoporated with a femtosecond laser line scan (5.8 µm in length) in the presence of propidium iodide (**Figure** **3**a). Successfully optoporated cells showed propidium iodide uptake (Figure 3b–d). When optoporating without nanoparticles, propidium iodide uptake with ≈2% efficiency only occurred at laser powers above 78 mW (Figure 3e). This value marked the energy threshold needed for unassisted optoporation, in agreement with the established range of 50–100 mW for unsensitised optoporation.^[^ ^58^ ^]^ In contrast, nanoparticles enhanced laser coupling as propidium iodide uptake achieved 43% efficiency at powers of 65 mW. However, lowering laser energy also reduced optoporation efficiency, dropping delivery efficiency to 5% at 39 mW (Figure 3e). As there was no difference in delivery efficiency between nanodisks and nanorods, we opted to use nanodisks moving forward, due to their higher production yield. Taken together, these results demonstrate the feasibility of using porous silicon nanoparticles for spatially‐resolved optoporation.

To optimize delivery efficiency, we assessed propidium iodide uptake as a function of nanoparticle concentration between 30 and 150 µg mL^−1^ and laser powers between 20 and 65 mW. Overall, the delivery efficiency increased as the nanoparticle concentration increased. In the presence of 100 µg mL^−1^ nanoparticles, propidium iodide uptake was at 22% at 29 mW, increasing to 43% at 52 mW, then rising further to 62% at 65 mW (Figure 3f). To further investigate optoporation parameters, we selected 29 and 65 mW as power values representative of the range of achievable delivery efficiencies.

High‐repetition femtosecond lasers operating at MHz range can accumulate heat from overlapping pulses, potentially causing cell damage.^[^ ^59^ ^]^ To minimize exposure time and resultant thermal energy while preserving delivery efficiency, we explored the role of line scanning speed between 8.4 and 336 µm s^−1^ while quantifying propidium iodide delivery. Scanning speed had no impact on delivery efficiency regardless of nanoparticle concentration or laser power (Figure 3g,h). Based on these results, we selected 336 µm s^−1^ as a means to minimize exposure time and thus potential damage to the cell without compromising delivery efficiency. Additionally, higher speeds enabled higher throughput due to reduced scanning time. Although the 150 µg mL^−1^ concentration showed superior performance compared to the 100 µg mL^−1^ concentration, this higher concentration hindered cell visibility, complicating the targeting of individual cells (Figure S3, Supporting Information). Therefore, we opted to proceed with the investigation using a nanoparticle concentration of 100 µg mL^−1^.

### 2.4 Nanoparticles for mRNA Transfection

**Figure 4:**
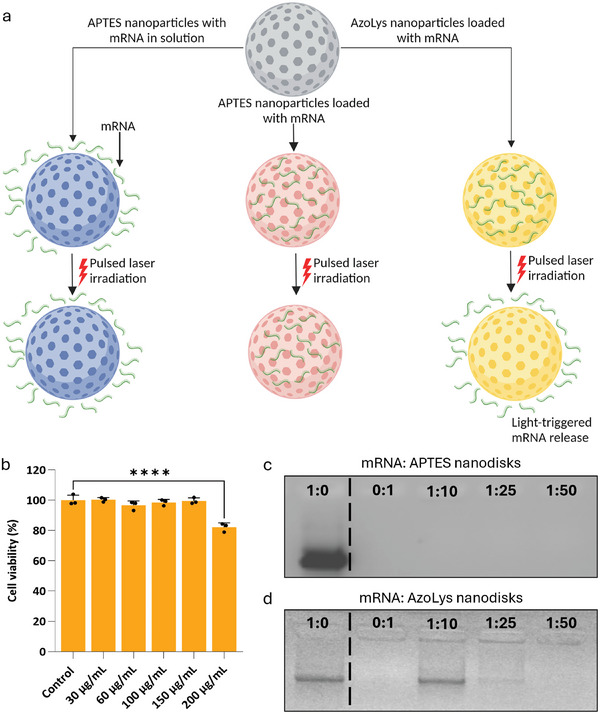
Porous silicon nanoparticles for mRNA transfection. a) Schematic illustration of APTES nanoparticles with mRNA in solution, APTES nanoparticles loaded with mRNA, and AzoLys nanoparticles loaded with mRNA displaying the light‐triggered mRNA release. Created with BioRender.com. b) Cell viability assay examining cytotoxicity of AzoLys nanodisks for MCF‐7 cells as a function of nanodisk concentration. Viability is expressed relative to untreated control. Data was collected from *N* = 3 biological replicates. Statistical analysis by ordinary one‐way ANOVA test with Tukey's post‐hoc test was performed to determine a significant difference (****p < 0.0001). Data shown as mean with ± standard deviation. c,d) Gel electrophoresis assay for mRNA complexation with c) APTES and d) AzoLys nanoparticles.

We evaluated different methods for spatially‐resolved mRNA transfection. In one method, we simply dispersed mRNA in the cell culture during optoporation (**Figure** **4**a). Alternatively, we explored ways to accumulate mRNA in proximity to the cells. In this approach, we complexed the negatively charged mRNA to the positively charged APTES nanoparticles. In a third approach, we used nanoparticles functionalized with a photo‐switchable molecule, ICPES–azobenzene–lysine (AzoLys), with a surface charge of 42.0 mV ± 2.6. Like the APTES nanoparticles, these AzoLys nanoparticles could bind mRNA through electrostatic interactions. Additionally, they could release mRNA during optoporation due to trans‐to‐cis isomerisation when exposed to near‐infrared (NIR) light^[^ ^43^, ^60^ ^]^ (Figure 4a). Both APTES and AzoLys nanoparticles exhibited good cytocompatibility at concentrations up to 200 µg mL^−1^ (Figure 4b).

We assessed the mRNA loading of APTES and AzoLys nanoparticles at three weight ratios, namely 1:10, 1:25, and 1:50 (mRNA: nanoparticles) by gel electrophoresis. APTES nanoparticles did not show a visible band of uncomplexed mRNA at any of the tested weight ratios (Figure 4c). AzoLys nanoparticles showed a strong band of uncomplexed mRNA at a 1:10 ratio, a faint band at a 1:25 ratio, and no visible band at a 1:50 ratio (Figure 4d). Moving forward, we opted for the 1:25 complexation ratio to achieve maximal complexation with minimal mRNA waste for both conditions.

### 2.5 mRNA Release Profile

We characterized the mRNA release profile from APTES and AzoLys nanoparticles (Figure S4a, Supporting Information). mRNA‐loaded nanoparticles were subjected to laser irradiation at 20, 40, or 70 mW for 10 min (12, 24, and 42 J respectively), or 70 mW for 30 min (126 J). APTES nanoparticles exhibited negligible mRNA release across all the tested energies, retaining the payload across all stimulation conditions. In contrast, AzoLys nanoparticles demonstrated a dose‐dependent mRNA release. To assess the potential for repeated dosing, we performed consecutive exposures on the same sample (Figure S4b, Supporting Information). Analogous to the dose‐response experiment, APTES nanoparticles showed negligible mRNA release. In contrast, AzoLys nanoparticles exhibited a release of 3.7% of mRNA upon initial exposure, but no detectable release in subsequent exposures, suggesting that the release mechanism is not amenable to multiple triggers. Potential mRNA leaching prior to laser irradiation, was evaluated by monitoring mRNA release from APTES and AzoLys nanoparticles in solution over a 24 h period (Figure S4c, Supporting Information). Both nanoparticle types demonstrated minimal spontaneous mRNA release, suggesting robust payload retention in the absence of laser stimulation. These findings collectively indicate that mRNA release from AzoLys nanoparticles is primarily laser‐triggered, occurs as a single event, and resists leaching under physiological conditions.

### 2.6 Optoporation Impact on Cells

**Figure 5:**
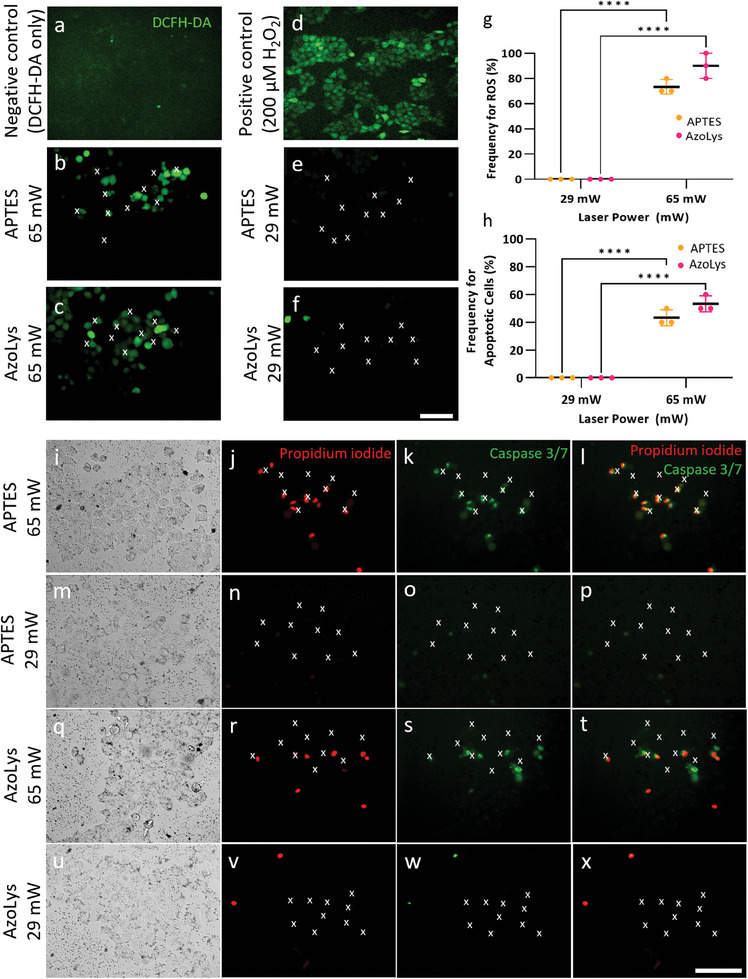
Impact of optoporation on cells. a–f) Fluorescence microscopy images of DCFH‐DA staining showing ROS generation within cells. a) Untreated control cells, b,c) cells optoporated at 65 mW, with 100 µg mL^−1^ b) APTES and c) AzoLys nanoparticles, d) positive 200 µM H_2_O_2_ control, e,f) cells optoporated cells at 29 mW, with 100 µg mL^−1^ e) APTES and f) AzoLys nanoparticles. g) Quantitative analysis of the data shown in (a–f). Data was collected from *n* = 3 biological replicates. Statistical analysis by ordinary two‐way ANOVA with Tukey's post‐hoc test was performed to determine significant differences (****p < 0.0001). h) quantitative analysis of the caspase 3/7 activation data shown in (i‐x). Data was collected from *n* = 3 biological replicates. Statistical analysis by ordinary two‐way ANOVA with Tukey's post‐hoc test was performed to determine significant differences (****p < 0.0001). Data is shown as mean with ± standard deviation. i–x) Fluorescence microscopy images of cell death (propidium iodide red signal) and caspase 3/7 activation (green signal) for laser‐irradiated cells at i–l) 65 mW and q–t) 29 mW with 100 µg mL^−1^ APTES functionalised nanoparticles. m–p) Laser‐irradiated cells at 65 mW and u–x) 29 mW with 100 µg mL^−1^ AzoLys nanoparticles. Scale bars: 100 µm. White x marks indicate optoporation target cells.

Femtosecond laser irradiation can generate low‐density electron plasma and substantially increase intracellular reactive oxygen species (ROS).^[^ ^61^ ^]^ Although ROS are important signaling molecules that regulate proliferation, excessive accumulation causes oxidative stress and cellular damage.^[^ ^62^ ^]^ Therefore, ROS imaging with a small‐molecule fluorescent probe 2′,7′‐dichlorodihydrofluorescein diacetate (DCFH‐DA) provided early evidence of an unfavorable cellular environment (**Figure** **5**a–g). Optoporation at 65 mW induced ROS within 2 min in 73% of cells when using APTES nanoparticles (Figure 5b) and 90% of cells when using AzoLys nanoparticles (Figure 5c), analogously to ROS‐inducing H_2_O_2_ exposure (Figure 5d). In contrast, optoporation at 29 mW did not exhibit ROS generation for either APTES or AzoLys nanoparticles, even after a 10 min incubation period (Figure 5e,f,g), analogously to untreated controls (Figure 5a).

Since the accumulation of ROS within cells can lead to oxidative stress and potentially trigger apoptotic cell death processes,^[^ ^63^ ^]^ we probed the apoptotic markers caspase 3/7 to assess the level of apoptosis (Figure 5h–x). Irradiating the cells with 65 mW at 100 µg mL^−1^ induced caspase 3/7 activation for 43% of cells when using APTES nanoparticles, and 53% when using AzoLys nanoparticles. Subsequently the same cells also tested positive for irreversible loss of membrane integrity, indicating a progression toward cell death (Figure 5i–l, q–t). On the other hand, no apoptotic cells or irreversible loss of membrane integrity were detected for optoporation at 29 mW with 100 µg mL^−1^ nanoparticles (Figure 5m–p, u–x).

We also investigated the impact of optoporation on cell proliferation. Cells were exposed to 100 µg mL^−1^ of either APTES or AzoLys nanoparticles and subjected to laser irradiation. Cells exposed to the laser without nanoparticles and cells exposed to nanoparticles without laser irradiation served as controls. The following day, cells were fixed and stained for Ki‐67, a marker of cellular proliferation. The proliferation index showed an analogous proliferation for treated and control cells (Figure S5, Supporting Information).

### 2.7 mRNA Transfection by Spatially‐Resolved Optoporation

**Figure 6:**
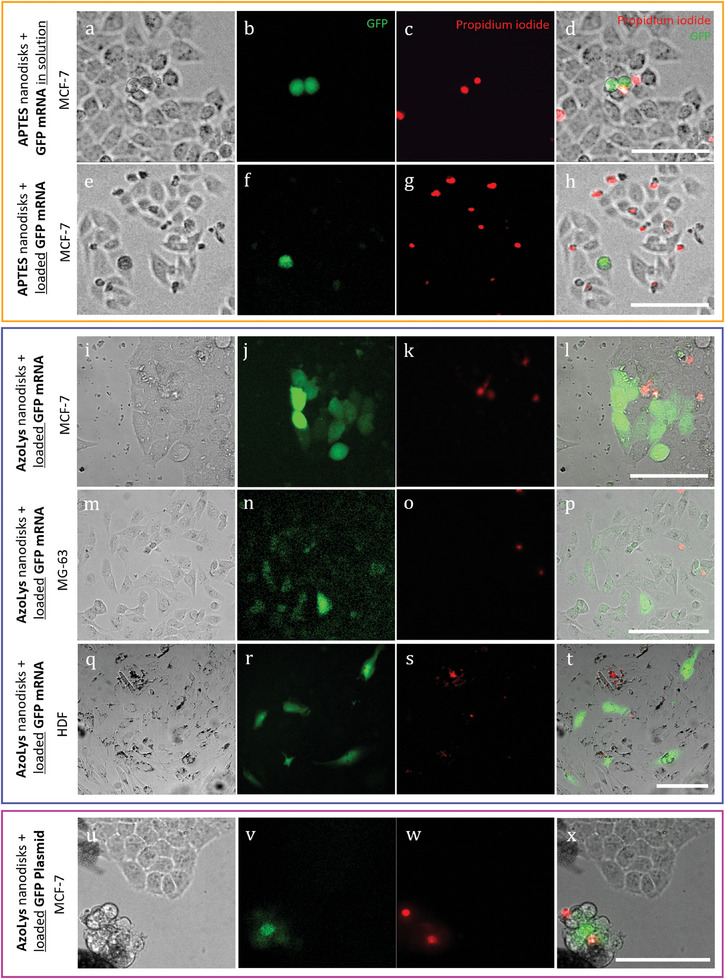
Spatially‐resolved mRNA transfection in 2D. a) Bright field and b,c) fluorescence microscopy images of MCF‐7 cells 24 h following spatially‐resolved mRNA transfection by optoporation using mRNA in solution. b) GFP expression, c) cell death, d) overlay of (a–c). e) Bright field and f,g) fluorescence microscopy images of MCF‐7 cells 24 h following spatially‐resolved mRNA transfection by optoporation using APTES nanoparticles loaded with mRNA. f) GFP expression, g) cell death, h) overlay of (f,g). i–t) microscopy images of MCF‐7, MG‐63, and HDF cells 24 h following spatially‐resolved mRNA transfection by optoporation using AzoLys nanoparticles loaded with mRNA. j,n,r) GFP expression, k,o,s) cell death, and l,p,t) overlays. u) Bright field and v,w) fluorescence microscopy images of MCF‐7 cells 48 h following spatially‐resolved GFP‐plasmid transfection by optoporation using AzoLys nanoparticles loaded with GFP‐plasmid. Scale bar: 100 µm.

To evaluate the efficiency of spatially‐resolved mRNA transfection, we first optoporated a 2D culture of MCF‐7 cells. Using mRNA in solution, optoporation in the presence of 100 µg mL^−1^ of APTES nanoparticles could selectively transfect cells with eGFP, however only achieving ≈2% efficiency (**Figure** **6**a–d). The complexation of mRNA to the APTES nanoparticles in analogous conditions did not yield significant improvement in efficiency (Figure 6e–h). On the contrary, in the same conditions, AzoLys nanoparticles loaded with mRNA achieved 80% transfection efficiency (Figure 6i–l; Figure S6a,b, Supporting Information). Quantified transfection efficiency (median) is summarised in Figure S7 (Supporting Information). While the performance of APTES nanoparticles was deemed insufficient to support reliable spatially‐resolved transfection, the high efficiency of AzoLys nanoparticles compared favorably with literature reports of 2D optoporation using established nanomaterials.^[^ ^23^, ^24^, ^25^, ^32^, ^64^, ^65^ ^]^ To evaluate optoporation effectiveness across cell types we monitored the delivery and expression of GFP mRNA in the human osteosarcoma cell line MG‐63 (Figure 6m–p) as well as primary human dermal fibroblasts (Figure 6q–t). Confocal images of GFP transfection in HDF cells are shown in Figure S6c,d, Supporting Information). We also tested optoporation of a GFP‐plasmid construct. These experiments demonstrated the broad applicability of optoporation with AzoLys nanoparticles, as evident from the detectable expression of the GFP reporter (Figure 6u–x).

**Figure 7:**
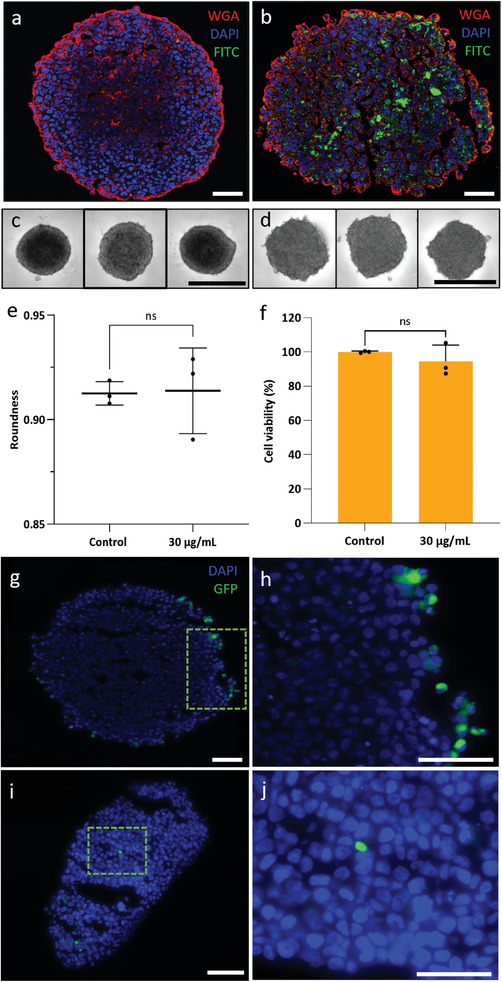
Spatially‐resolved mRNA transfection in organoids. a,b) Fluorescence microscopy images of histological cross sections of an MCF‐7 Spheroid a) formed without nanodisks (control) and b) with 30 µg mL^−1^ of FITC‐tagged nanodisks. Images show the nanodisks distribution across the volume of the spheroid. Scale bar: 100 µm. c,d) Bright‐field microscopy images of spheroids formed c) without or d) with 30 µg mL^−1^ nanodisks following incubation for 24 h in a hanging drop. Scale bar: 500 µm. e) Comparison of the roundness of spheroids with and without nanodisks, analyzed from the data shown in (c,d). f) Cell viability assay examining cytotoxicity of nanodisks for MCF‐7 spheroids as a function of nanodisk concentration. Viability is expressed relative to an untreated control. Data was collected from *n* =  3 biological replicates. Statistical analysis by ordinary one‐way ANOVA with Tukey's post‐hoc test was performed to determine significant differences (*p < 0.05). Data is shown as mean with ± standard deviation. g,j) GFP expression in the spheroid after localized optoporation. The areas within the green boxes in (g,i) are shown in (h,j). Scale bar: 100 µm.

These results prompted us to further investigate the use of AzoLys nanoparticles for spatially‐resolved mRNA delivery into an MCF‐7 breast cancer spheroid, as an example of a 3D organotypic model. The nanoparticles were incubated with cells for 24 h during spheroid formation to facilitate uniform dissemination throughout the spheroid (**Figure** **7a,b**). The nanoparticle treatment did not affect the viability of the spheroids, and their overall morphology was preserved (Figure 7c–f). Optoporation targeting a sector of cells either on the surface of the spheroid or within its core achieved spatially‐resolved transfection, leading to localized GFP expression (Figure 7g–j). Controls included optoporation in the absence of the nanoparticles, and exposure of the spheroid to nanoparticles without optoporation, which did not yield GFP expression (Figure S8, Supporting Information). These data indicate that porous silicon nanoparticles can mediate spatially‐resolved transfection using optoporation in 2D and 3D organotypic models.

## 3 Conclusion

Our results show that biocompatible and bioresorbable porous silicon nanoparticles are capable of spatially‐resolved transfection of organoids. We identified the conditions to facilitate efficient intracellular delivery without inducing reactive oxygen species or compromising cell viability, enabling transient membrane permeabilization across a range of laser powers, scanning speeds, and particle concentrations. Efficient transfection requires the mRNA cargo to quickly and effectively diffuse into the cell cytosol during the transient increased membrane permeability induced by optoporation.^[^ ^66^ ^]^ Therefore, alongside the use of mRNA in solution, we explored strategies to concentrate mRNA at the site of optoporation, by complexing with APTES and AzoLys positively‐charged nanoparticles. AzoLys further provided light‐induced mRNA release. The mRNA concentration and triggered release of AzoLys were essential to achieve spatially‐resolved transfection at a efficiencies as high as 84%. The AzoLys nanodisks successfully mediated spatially‐resolved transfection of 3D MCF‐7 spheroids, allowing for localized expression both on the surface and within the core of the spheroids.

This study presents a versatile platform for precise genetic manipulation by combining the unique properties of porous silicon nanoparticles with optimized surface functionalization and two‐photon excitation. The ability to achieve spatially‐controlled transfection in 3D cellular systems opens new avenues for cancer research to study dynamic changes in tumor microenvironments and to model and dissect the stages of human development. This ability to achieve spatially‐resolved transfection in organoid models opens new possibilities for controlled gene expression, enhancing the flexibility and representativeness of engineered biological systems for the study of human health.

## 4 Experimental Section

### Nanoparticle Fabrication through Electrochemical Etching

Porous silicon films were anodised from 100 mm boron‐doped p+ type Si (100)‐oriented wafers (University Wafers Inc, USA) with a resistivity of 0.01–0.02 Ω cm in an electrolyte solution of hydrofluoric acid (HF, 50%, VLSI Selectipur, 7664‐39‐3) and absolute ethanol (99%, Sigma–Aldrich, 32221) in a 1:2 volumetric ratio. Electrochemical etching of porous multilayers was carried out in a custom‐made Teflon etch cell with the backside of the wafer in contact with an aluminum sheet and the front side of the wafer exposed to the anodizing electrolyte. A platinum mesh was used as the counter electrode in the solution. Each etching cycle consisted of two current densities applied successively: 42 mA cm^−2^ for 3 s, followed by 168 mA cm^−2^ for 0.35 s. The cycle was repeated 360 times, and then, a lift‐off layer was generated using 246 mA cm^−2^ for 1 s. The etched wafer was heated in air at 300 °C for 2 h.

The porous silicon films were then scrapped from the wafer and fractured by probe sonication (Sonics & Materials™ Ultrasonic Processor model VCX130) in isopropanol (IPA, Sigma–Aldrich, 59300) for 6 h, 90 W with 4 s on and 4 s off. The resulting dispersion of nanoparticles was centrifuged for 5 min at 20,000 rcf and the pellet was redispersed in IPA via sonication for a few seconds. The resulting dispersion was centrifuged at 1300 rcf for 5 min and the supernatant solution containing the particles of the desired size was collected. This step was repeated 5 times until the supernatant was almost clear.

### Nanoparticle Fabrication through Metal‐Assisted Chemical Etching

The silicon wafer was dipped in a solution of 5 mL 0.4 silver nitrate (AgNO_3_, Sigma–Aldrich, 31630), 20 mL HF, and 75 mL of distilled water for 2 min with continuous mixing. The wafer was then washed with water and ethanol and left to dry. Next, the wafer was dipped in 400 mL of etching solution H_2_O_2_: HF (1.5% v/v) for 20 min. The etching solution was prepared by mixing 80 mL of 49% v/v HF, 6 mL 30% wt hydrogen peroxide (H_2_O_2_, Acros Organics, AC411885000), and topped up with distilled water. To stop the etching, the wafer was removed from the etching solution, rinsed with distilled water and ethanol then dried with compressed air. The silver was removed by dipping the etched wafer in gold etchant (Sigma–Aldrich, 651818) solution for 10 min. The wafer was then washed and left to dry. The etched wafer was heated in air at 300 °C for 2 h. The etched silicon layer was scrapped off from the wafer and fractured by ultrasonication (Elma, model Elmasonic S) in water for 8 h. The resulting dispersion was centrifuged at 3000 rcf for 5 min and the supernatant solution containing the particles of the desired size was collected.

### Amine Group and Fluorescent Functionalisation of Porous Silicon Nanoparticles

Functionalization involved mixing 1 mg mL^−1^ of nanoparticles with a 2% 3‐aminopropyltriethoxysilane (APTES, Thermo Scientific, 430941000) in ethanol, and incubating the mixture in a thermoshaker (Eppendorf Thermomixer C Model 5382) at 800 rpm at room temperature for 2 h. The resulting functionalized particles were washed twice with IPA, once with ethanol, and redispersed in 4 mL of ethanol. Fluorescein isothiocyanate (FITC, Sigma–Aldrich, F7250, 0.0213 mmoles) was added with APTES tagged nanoparticles (3 mg) and left mixing in a thermoshaker at 800 rpm for 2 h at room temperature. To stop the functionalization reaction, the particles were washed three times with ethanol, redispersed in a known volume of ethanol, and stored at 4 °C for later use.

### Particle Size and Surface Charge Measurements

Particle size was characterized by dynamic light scattering (DLS) measurements (Zetasizer Nano ZS, Malvern Instruments, Malvern, U.K). Readings of the hydrodynamic diameter and polydispersity index (PDI) were done at 25 °C with 1.5 mg mL^−1^ nanoparticle concentration in ethanol with viscosity set to 1.07 cP and refractive index to 1.36. Measurements were taken three times and average values were reported.

Surface charge was characterized by ζ‐potential measurements (Zetasizer Nano ZS, Malvern Instruments, Malvern, U.K). The electrophoretic mobility of porous silicon nanoparticles was measured via the electrophoretic light scattering (ELS) technique using the Schmolukowski equation. The measurements were performed by dispersing the nanoparticles in ethanol. Measurements were taken three times and average values were reported.

### Brunauer–Emmett–Teller (BET) Analysis

Nitrogen adsorption–desorption isotherms of the porous silicon nanoparticles were recorded at 77 K using a micromeritics ASAP 2020 volumetric apparatus. Prior to the adsorption experiment, the samples were outgassed overnight in situ at 303 K. The pore diameter was determined using the BJH (Barrett, Joyner, and Halenda) method whilst the surface area of the sample was determined from the BET (Brunnauer–Emmett–Teller) theory.

### Cell Culture

Michigan Cancer Foundation‐7 (MCF‐7) epithelial breast cancer cell line were cultured in Dulbecco's Modified Eagle Medium (DMEM), high glucose, GlutaMAX (Gibco, 10569010) supplemented with 10% fetal bovine serum (FBS) (Gibco, 10270106) and 1% Penicillin‐Streptomycin (P/S, Gibco, 15070063). Cells were seeded at 60,000 cells per well for 24 h at 37 °C, 5% carbon dioxide in humidified incubators before optoporation.

### Cell Preparation for SEM

On a 13 mm glass coverslip (VWR, 631‐0148) 60,000 MCF‐7 cells were seeded and incubated for 24 h. After 24 h, the desired concentration of nanoparticles in OptiMEM was added and left incubating for a further hour followed by washing and fixation. Alternatively, excess nanoparticles were washed after 1 h of incubation and the cells were incubated for an additional 24 h followed by fixation. The cells were fixed with 4% wt/vol paraformaldehyde (PFA) for 15 min and then washed three times in phosphate buffer saline (PBS, Sigma–Aldrich, D8537) at 5 min intervals. Subsequently, the cells were dehydrated through a graded ethanol series starting with 50% ethanol for 10 min, then increasing the ethanol concentration to 75, 90, 95, and 100%, each time leaving the solution for 5 min. The 100% ethanol step was repeated twice, the second time left for 10 min. The cells were submerged in a solution of hexamethyldisilazane (HMDS, Sigma–Aldrich, 440191) and ethanol in the ratio of 1: 2 respectively which was then changed to 2: 1 respectively and finally to 100% HMDS, each step for 10 min and then left to dry overnight. The samples were sputter‐coated with an ≈10 nm thick layer of gold before SEM imaging. The SEM images in Figure 2b,c,e,f were processed with Adobe Photoshop to increase the contrast of the nanoparticles, allowing better visualization of the nanoparticles and the cells.

### Confocal Imaging for Endocytosis

Cells were fixed with 4% PFA, permeabilized with 0.25% (v/v) Triton X‐100 (Sigma Aldrich), stained with Alexa Fluor 555 Phalloidin and DAPI to visualize the cell cytoskeleton and nucleus, respectively. The cells were imaged in an eight‐well glass bottom chamber (ibidi) using a Zeiss LSM 980 confocal microscope.

### TEM Imaging

The nanoparticles were dispersed in ethanol at a concentration of 50 µg mL^−1^ and sonicated for 30 s to ensure a uniform suspension. A 4 µL aliquot of the nanoparticle suspension was deposited onto a carbon‐coated copper grid (Agar Scientific) and allowed to air dry. Excess solvent was carefully removed with filter paper. TEM imaging was performed using a JOEL JEM‐1400 Plus microscope at an accelerating voltage of 80 kV.

### CellTiter‐Glo Viability Assay in the 2D System

MCF‐7 cells were seeded at 10 000 cells per well in 96‐well plates in 100 µL supplemented DMEM and incubated for 24 h. Nanoparticle solutions were prepared in OptiMEM‐reduced serum media (Gibco, 11058021). The DMEM was removed from the cells, replaced with 100 µL of the nanoparticle solution, and incubated for a further hour. After 1 h, the porous silicon nanoparticles were gently washed away with 100 µL fresh DMEM, and cells were further incubated for 24 h. The viability of MCF‐7 was assessed for 24 h after exposure to porous silicon nanoparticles using CellTitre‐Glo 2.0 viability assay reagent (Promega, G924B) as recommended by the manufacturer. The luminescent signal of each well was measured using a CLARIOstar Plus plate reader (BGM lab tech) with a 3600 gain.

### Immunofluorescence

For the immunolabelling of Ki‐67 the samples were fixed in 4% ice‐cold PFA in PBS for 15 min at room temperature. After fixation, the samples were washed 3 times for 5 min each and permeabilized with Triton X at 0.1% in PBS for 15 min. The samples were washed a following 3 times for 5 min before blocking for 30 min at room temperature with Invitrogen IHC/ICC (Thermo Fisher Scientific, 00‐4952‐54) blocking buffer. The samples were then incubated with primary antibodies against Ki‐67 (Thermo Fisher Scientific, MA5‐41135) for 1 h in a blocking buffer at room temperature. The samples were washed 3 times with PBS for 5 min each before incubation of the secondary antibodies (Thermo Fisher Scientific, A‐31576) in blocking buffer for 1 h at room temperature. The samples were then stained with DAPI (Sigma Aldrich, D9542) in PBS for 10 min after a further round of PBS washes.

### Cell Proliferation Analysis

The nuclei of each cell from the images obtained for each sample were segmented using the nuclei model of Cellpose (2.2). The intensity of Ki‐67 images was measured using the nuclei masks and region properties function from scikit‐image (0.19.3). The intensity of Ki‐67 images was normalized to the negative control and the distribution of intensities was calculated. Cells with Ki‐67 intensities that fell within the first and third quantiles were counted. These counted Ki‐67 cells were taken as a ratio of the total counted nuclei of each corresponding image resulting in a proliferation index.

### MCF‐7 Spheroid Preparation

Cells were suspended in 1 mL of 1.2% w/v of Methocel A4M (3000–5000 mPa.s – medium viscosity, Sigma–Aldrich, 94378): DMEM with a final concentration of 1:4 (v: v) to form the seeding solution. Repeatedly, 25 µL containing 3000 cells were withdrawn from the seeding solution and transferred as individual drops to the lid of the petri dish. The bottom of the petri dish was covered with 5 mL of PBS to serve as a hydration chamber. The lid was inverted and placed on the top of the petri dish for the drop to hang. The cells were incubated for 24 h to form a spheroid.

### Addition of Nanoparticles to the Spheroids

The desired concentration of the nanoparticles was withdrawn, and centrifuged to form a pellet, and the supernatant was removed. The pellet was resuspended by sonicating with 100 µL of DMEM. To the nanoparticle suspension, cells, DMEM, and Methocel were added to form a seeding solution with a final concentration of 1: 4 (v: v) Methocel: DMEM. The resulting solution was used to form the individual drops.

### CellTiter ‐Glo Viability Assay in the 3D System

The spheroids were transferred with a pipette tip to a 96‐well plate where each well contained 4 spheroids to reduce variability per well. The viability was assessed for 24 h after exposure to porous silicon nanoparticles using CellTiter‐Glo 3D luminescent cell viability assay (Promega, G968A) as recommended by the manufacturer. The luminescent signal of each well was measured using a CLARIOstar Plus plate reader with a 3600 gain.

### Multiphoton Femtosecond Laser Set‐up

Optoporation and imaging of the cells were performed via a homemade setup. A 200 fs pulsed laser with tuneable wavelengths (700–1068 nm) pumped via a Verdi laser through an optical path reaching the inverted epifluorescent microscope (Nikon). All optoporation experiments were carried out at 800 nm and calibrated at the start of every experiment. The size and location of the areas targeted for optoporation, the scanning speed, and the laser power were controlled via a custom‐made micromanager controller system. Samples were illuminated through a 20x objective, 0.75 numerical aperture and the camera captured images automatically before and after optoporation.

### Cellular Optoporation Efficiency in 2D Cell Cultures

MCF‐7 cells were incubated at a seeding density of 40 000 in an ibidi‐glass bottom 8 well plates (Ibidi, 80807) for 24 h to achieve ≈60% confluency. After 24 h, the DMEM was replaced with an OptiMEM solution containing nanoparticles and incubated for a further hour. After 1 h, unbound nanoparticles were washed away with OptiMEM. The OptiMEM was replaced with a solution of OptiMEM containing 7.5 µm of propidium iodide (Sigma–Aldrich, P4864). Cells were optoporated at a range of laser powers and scanning speeds. A new field of view was obtained for each different laser power or scanning speed tested. The success of propidium iodide delivery was calculated by subtracting the signal of the pre‐optoporation from the post‐optoporation image and checking whether the propidium iodide inside the cells correlated with the same cells that were targeted with the laser. Three fields of view per experiment, per condition, were tested.

### Short‐Term Viability Post‐Optoporation

Immediately post‐optoporation, cell viability was probed with calcein‐acetomethoxy (AM) (2.5 µm, BD Bioscience, 564061). After 10 min the cells were washed with OptiMEM and imaged. Overall, each experiment had three replicas, with three fields of view per replica for every different condition tested.

### Imaging for ROS

Cells were incubated at a seeding density of 40 000 in an ibidi‐glass bottom 8 well plates (Ibidi, 80807) for 24 h and incubated with nanoparticles for an hour. The negative control involved adding 1 µm of fluorescent 2′,7′–dichlorofluorescein diacetate (DCFH‐DA, abcam, ab113851) and image after 10 min of incubation. For the positive control, 200 µm of H_2_O_2_ was incubated with the cells for 1 h followed by washing with OptiMEM and incubating DCFH‐DA for 10 min before imaging. In the optoporation experiment, DCFH‐DA was added to the cells after optoporation, and imaging was done at 2 and 10 min after optoporation.

### Imaging for Caspase 3/7

Cells were incubated at a seeding density of 40 000 in an ibidi‐glass bottom 8 well plates (Ibidi, 80807) for 24 h and incubated with nanoparticles for an hour. Cells were then optoporated in the presence of 5 µm caspase 3/7 (Invitrogen, C10723) followed by imaging 1 h after optoporation.

### mRNA Transfection by Optoporation

Cells were incubated at a seeding density of 40 000 in an ibidi‐glass bottom 8 well plates (Ibidi, 80807) for 24 h and incubated with nanoparticles for an hour. After 1 h, unbound nanoparticles were washed away and cells were incubated with OptiMEM for 10 min to minimize mRNA degradation.^[^ ^25^ ^]^ The OptiMEM wash was removed from the cells and replaced by 100 µL of OptiMEM containing 0.5 µg of eGFP mRNA (EZ Cap, R1016) and 0.5 µg of eGFP Cy5 tagged mRNA (EZ Cap, R1011). Cells were immediately optoporated, then supplemented with fresh DMEM, and returned to the incubator for 24 h before analyzing the mRNA expression.

### Synthesis and grafting of Isocyanopropyltriethyoxysilane (ICPES)‐azobenzene‐Lysine on nanoparticles—Coupling reaction between Boc‐lysine(Boc)‐OH and 4,4’‐diaminoazobenzene

The synthesis and grafting process was as previously described.^[^ ^44^ ^]^ In summary, hydroxybenzotriazole (115 mg, 0,851 mmol) and N,N'‐dicyclohexylcarbodiimide (175 mg, 0,848 mmol) were mixed in a solution of Boc‐Lysine(Boc)‐OH (264 mg, 0,5 mmol) in anhydrous DMF (5 mL) at room temperature under nitrogen flux. After 1 h, 4,4'‐diaminoazobenzene (150 mg, 0,707 mmol) was added to the reaction and mixed at room temperature overnight. The mixture was then diluted in brine and extracted with ethyl acetate. The obtained product was dried on magnesium sulfate and the solvent removed under reduced pressure. The residue was purified by column chromatography (dichloromethane: ethyl acetate (70%: 30%)) and dried to form the orange solid azobenzene‐Lysine(diBoc).

### Synthesis and grafting of Isocyanopropyltriethyoxysilane (ICPES)‐azobenzene‐Lysine on nanoparticles—Coupling reaction between azobenzene‐Lysine(diBoc) and 3‐ICPES

3‐ICPES (4,6 µL, 0,018 mmol) was added to a solution of azobenzene‐Lysine(diBoc) (10 mg, 0,018 mmol) in anhydrous THF (4 mL) and the mixture was stirred at 65 °C under reflux for 24 h. n‐Pentane (5 mL) was added to the resulting product forming an orange precipitate, the excess pentane was decanted and left to evaporate to form the orange solid ICPES‐azobenzene‐Lysine(diBoc).

### Synthesis and grafting of Isocyanopropyltriethyoxysilane (ICPES)‐azobenzene‐Lysine on nanoparticles—Grafting of ICPES‐azobenzene‐Lysine(diBoc)

The obtained product ICPES‐azobenzene‐Lysine(diBoc) was added to a suspension of nanoparticles in 3 mL of toluene and mixed at 50 °C for 18 h under nitrogen flux. The reaction was stopped as the nanoparticles were centrifuged for 5 min at 20 000 rcf and rinsed three times in absolute ethanol.

### Synthesis and grafting of Isocyanopropyltriethyoxysilane (ICPES)‐azobenzene‐Lysine on nanoparticles—Deprotecting ICPES‐azobenzene‐Lysine(diBoc)

For the deprotection of lysine, the nanoparticles were resuspended in 3 mL of dichloromethane, and 2 mL of trifluoroacetic acid was added dropwise to the solution. The reaction was then stirred at room temperature for 30 min. The reaction was stopped by centrifuging the nanoparticles for 5 min at 20 000 rcf and rinsed three times in absolute ethanol.

### mRNA Complexation

mRNA and nanodisks were prepared in the mass ratio of 1: 25 to a final volume of 22 µL. The mRNA and nanoparticle mixture was left incubating at 37 °C for 1 h for complexation.

### mRNA Release Profile

The release kinetics of APTES and AzoLys nanoparticles were investigated by exposing 50 µL of nanoparticles (1 mg mL^−1^) to varying energies (0–126 J) at 371 nm wavelength. After each irradiation, samples were centrifuged at 6000 rpm at 4 °C, and the supernatant was analyzed using Qubit RNA High Sensitivity (Invitrogen, Q32855).

Incremental release was assessed by exposing 50 µL of nanoparticles (1 mg mL^−1^) to 70 mW irradiation for 10 min. The supernatant was collected after centrifugation and measured for mRNA. The same sample was resuspended in 50 µL of Citrate Buffer (0.1 m, pH 6.0) and re‐exposed to 70 mW for 10 min (42 J). This process was repeated a third time.

To evaluate spontaneous mRNA release, APTES and AzoLys nanoparticles were complexed with mRNA and incubated at room temperature for 24 h. mRNA release was quantified using Qubit the following day.

### Cellular Optoporation Efficiency in 3D Spheroids

A solution of 0.5 µg of eGFP mRNA and 0.5 µg of eGFP Cy5 tagged mRNA was diluted in 100 µL of OptiMEM and added with the spheroids. Optoporation was performed at multiple areas within the spheroid. After laser treatment, the cells were supplemented with fresh DMEM and returned to the incubator for 24 h before analyzing the mRNA expression.

### Spheroids Cryosections

The spheroids were fixed with 4% wt/vol PFA for 30 min and washed three times in PBS at 5 min intervals. Spheroids were left for 3 h in a 15% sucrose solution (Sigma–Aldrich, S9378) at 4 °C, then exchanged for a 30% sucrose solution and left overnight at 4 °C. The spheroids were embedded in O.C.T (CellPath, KMA‐ 0100‐00A) and the sections were collected with a cryotome (Bright, OTF5000). The chamber and specimen temperature was set at −20 °C and the 10 µm thick sections were collected on superfrost® plus slides (Fisher Scientific, 12625336).

### Statistical Analysis

All data were presented as mean with standard deviation and analyzed using the GraphPad Prism 9.4.1 software (La Jolla, CA, USA). The statistical tests used in each figure are mentioned in the figure caption. A *p*‐value <0.05 was set as the level of statistical significance.

## Conflict of Interest

The authors declare no conflict of interest.

## Author Contributions

C.S. performed methodology, investigation, formal analysis, project administration, wrote the original draft. V.C. performed investigation, methodology, and formal analysis. Y.W. performed investigation, methodology, and formal analysis. S.D.‐G. performed investigation, methodology, and formal analysis. M.K. performed investigation, methodology, and formal analysis; C.W., D.A.M., P.V., S.M.L. performed investigation; M.G.B. performed methodology. C.N., M.B., J.‐O.D. and F.C. performed methodology. C.C. conceptualized the project, performed methodology, formal analysis, and project administration, acquired resources and funds, wrote the original draft, and supervised the project.

## Supporting information



Supporting Information

## Data Availability

The data that support the findings of this study are available from the corresponding author upon reasonable request.
