# Supplementary material for: Spatially‐Resolved Organoid Transfection by Porous Silicon‐Mediated Optoporation
Source: Adv Mater. 2024 Oct 17;36(49):2407650. doi: 10.1002/adma.202407650 (PMC11619231; doi:10.1002/adma.202407650)
Supplement: Supplementary file 1 — Supporting Information [file ADMA-36-2407650-s001.docx]

**Supplementary Information**


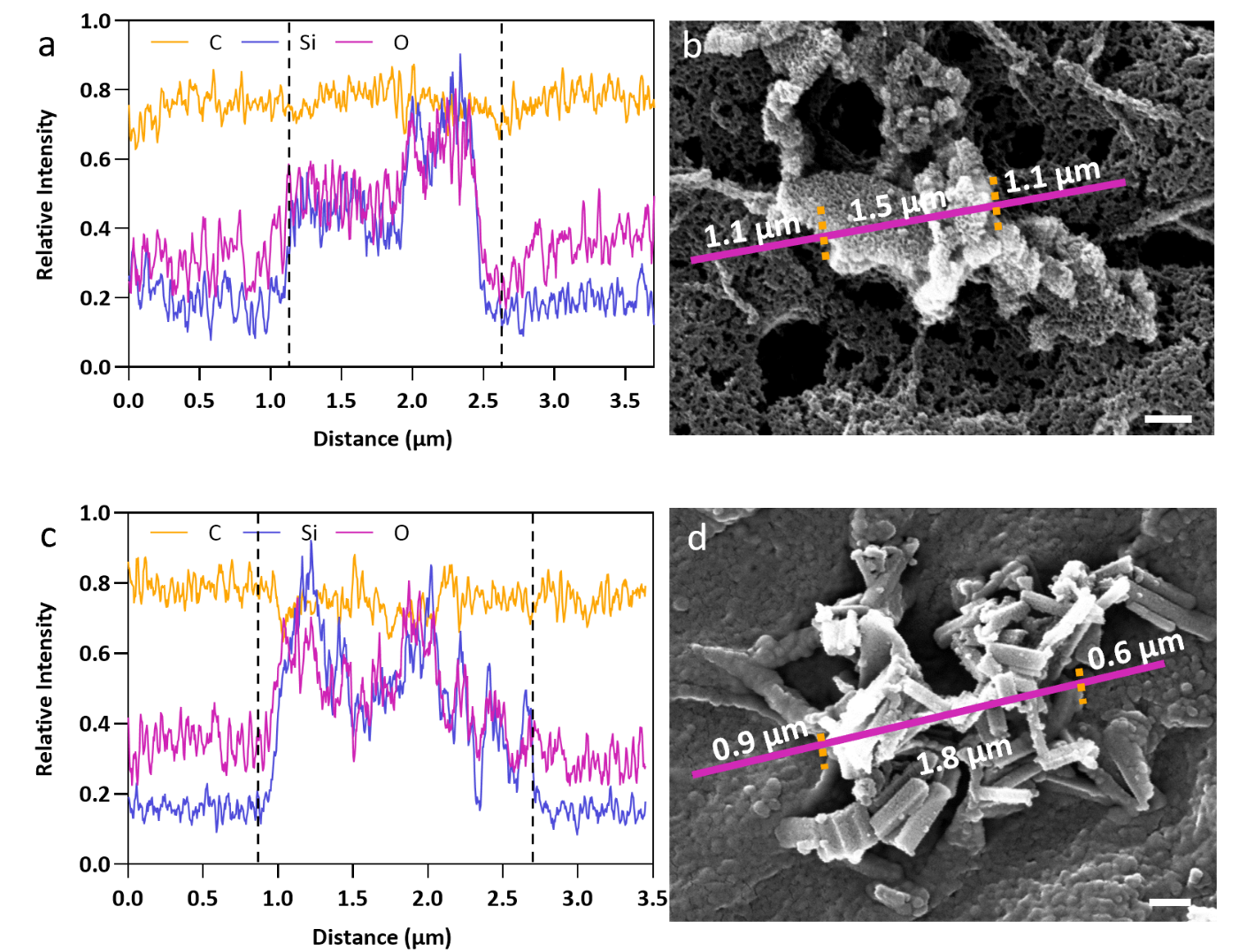


**Figure S1:** **EDX analysis and SEM imaging of nanodisks and nanorods on MCF-7 cells.**

**(a, c)** Elemental peak mapping of carbon (yellow), silicon (blue) and oxygen (magenta) and **(b, d)** show the region of the EDX line scan. Scale bar: 300 nm


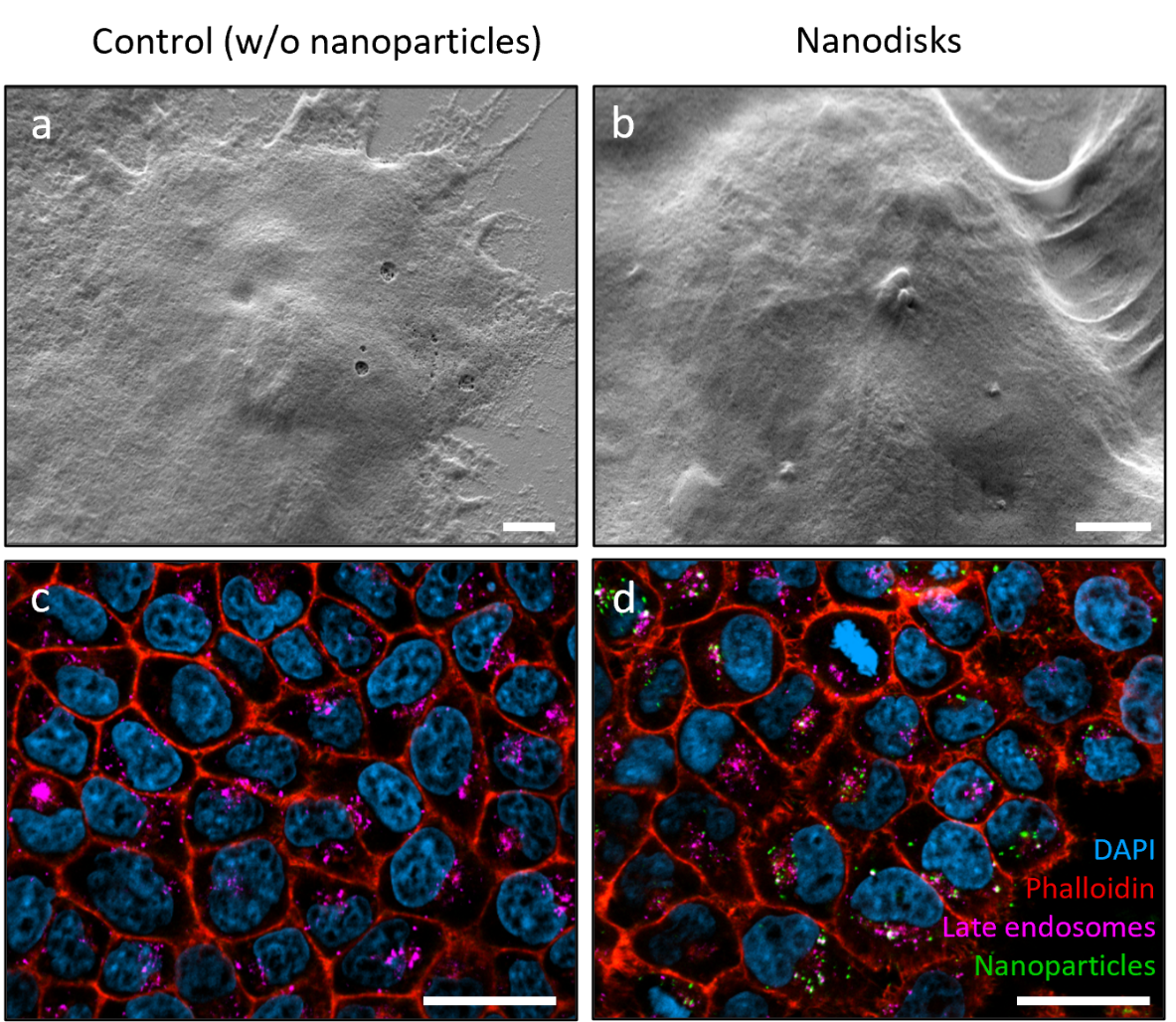


**Figure S2: SEM and confocal images of the MCF-7 with and without nanoparticles. (a)** SEM imaging of MCF-7 cells without nanoparticles, **(b)** MCF-7 cells after 24 hour incubation with 30 µg/mL of nanodisks. **(c)** Confocal imaging of MCF-7 cells without nanoparticles, **(d)** with nanoparticles after 24 hour incubation with nanodisks. Scale bars: **(a,b)** 2 µm, **(c,d)** 50 µm.


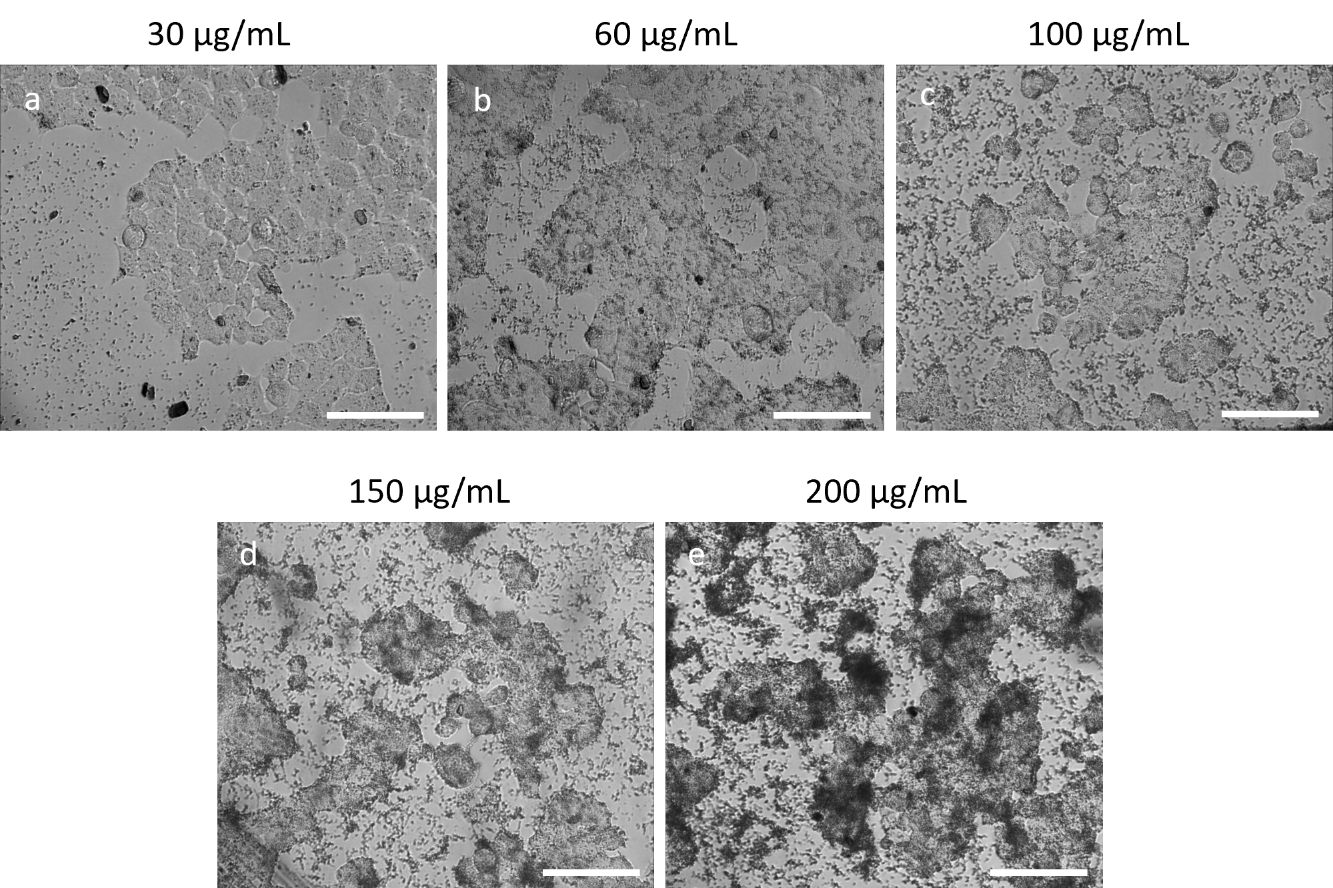


**Figure S3: Co-incubation of nanodisk nanoparticles with MCF-7 cells**. **(a-e)** Bright-field images after the addition of different concentration of nanodisks with the MCF-7 cells followed by washing of the unbound nanoparticles after 1 hour of incubation. Scale bar: 100 μm


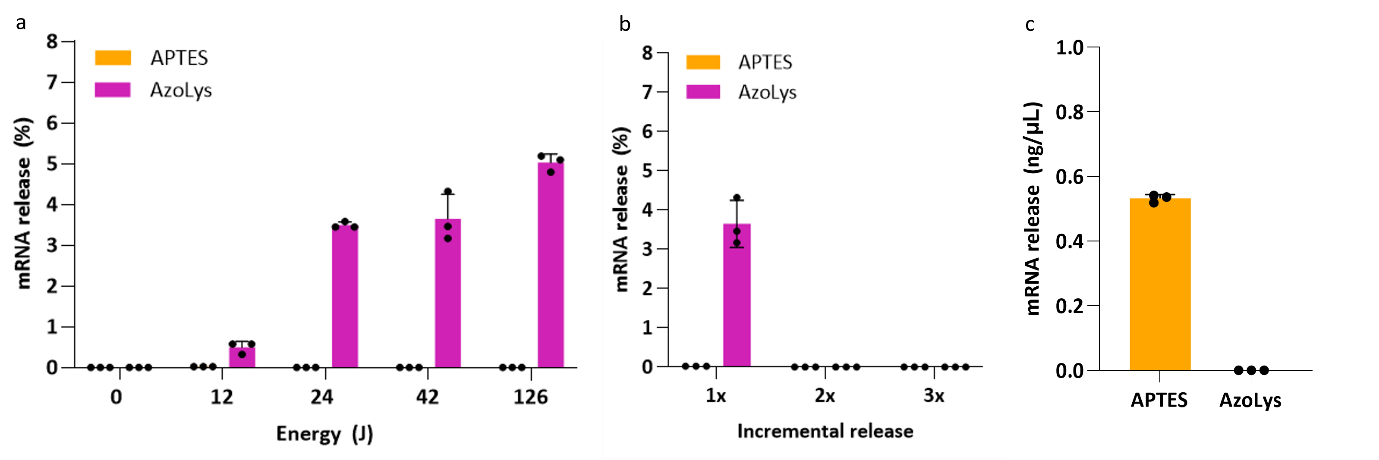


**Figure S4:** **mRNA release profiles from APTES and AzoLys nanoparticles under various conditions. (a)** Release kinetics of mRNA from APTES and AzoLys nanoparticles under different laser powers and exposure times. **(b)** Consecutive laser exposures at 42 J demonstrating single-event release from APTES and AzoLys nanoparticles, where 1x represents the initial exposure, 2x represents the second exposure, 3x represents the third exposure. **(c)** Comparison of mRNA leaching from APTES and AzoLys nanoparticles over 24 hours after loading without laser stimulation. Data was collected from N=3 replicates, data shown as mean with ± standard deviation. Detection limit of Qubit™ RNA High Sensitivity is 0.2 ng/μL.


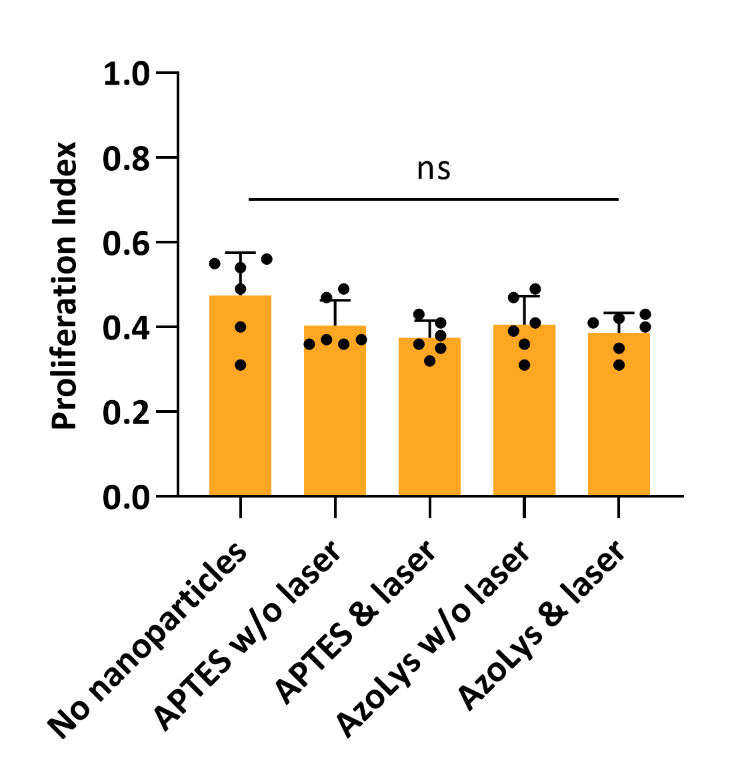


**Figure S5: Effect of optoporation on MCF-7 cell proliferation.** Ki-67 staining of MCF-7 cells under various conditions: no nanoparticles (control), APTES nanoparticles (100 μg/mL) without laser irradiation (negative control), APTES nanoparticles with laser irradiation, AzoLys nanoparticles (100 μg/mL) without laser irradiation (negative control), and AzoLys nanoparticles with laser irradiation. Data was collected from N=3 biological replicates with two technical repeats per replicate. Statistical analysis by one-way ANOVA with Tukey’s post-hoc test was performed to determine significant differences. Data shown as mean with ± standard deviation.


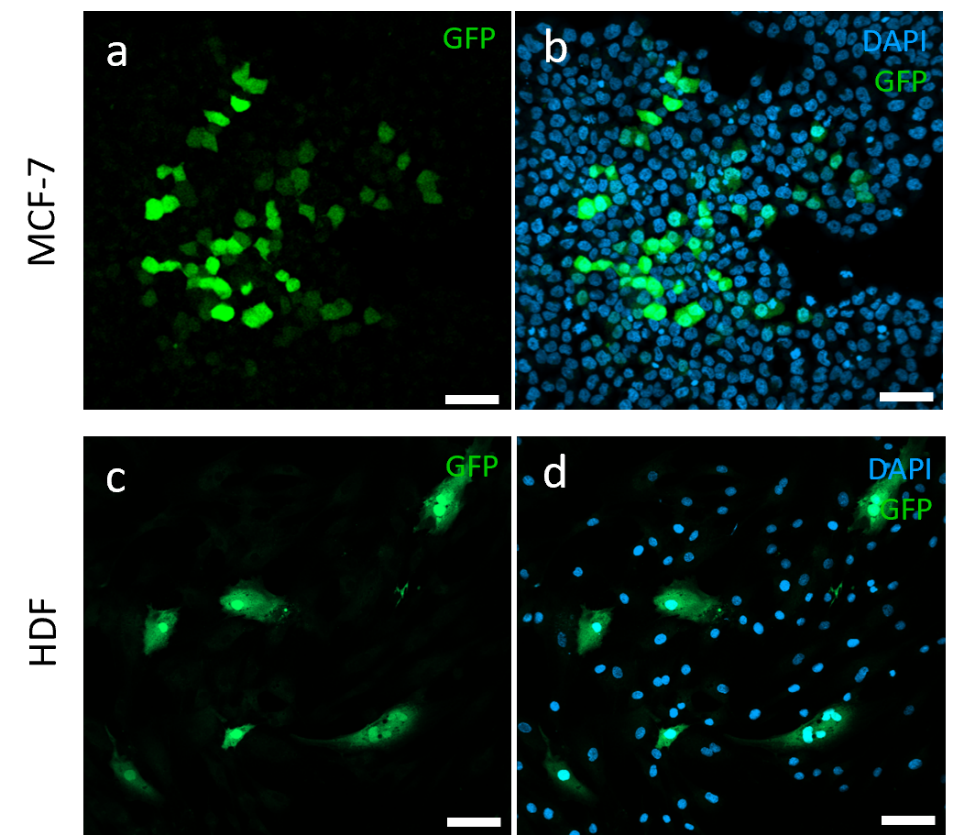


**Figure S6: Confocal images of the GFP transfection in MCF-7 and HDF cells using AzoLys nanoparticles.** **(a,b)** GFP expression in MCF-7 cells after optoporation and **(c,d)** GFP expression in HDF primary cells after optoporation. Scale bar: 100 μm.


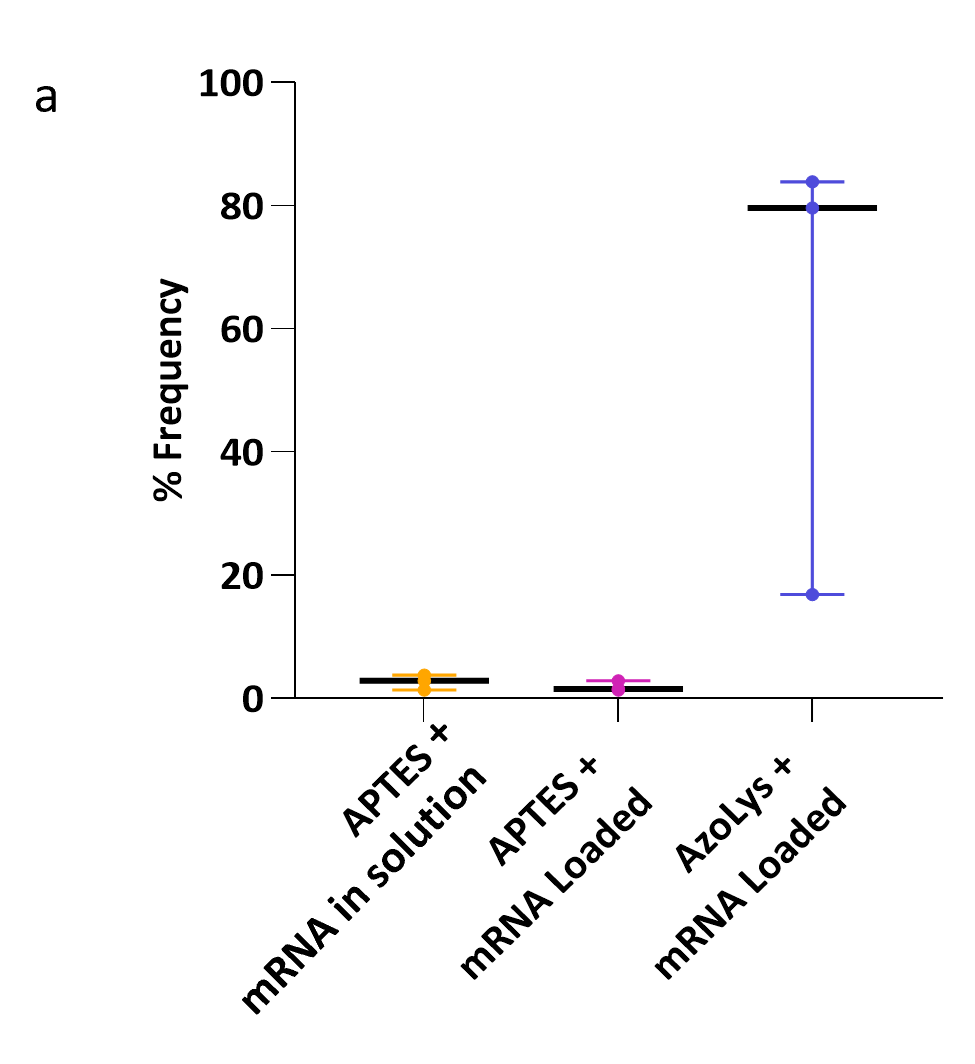


**Figure S7: Efficiency of GFP mRNA transfection using APTES and AzoLys nanodisks with MCF-7 cells**. **(a)** Quantification of transfection efficiency. Data shown as median with range, N=3 independent experiments.


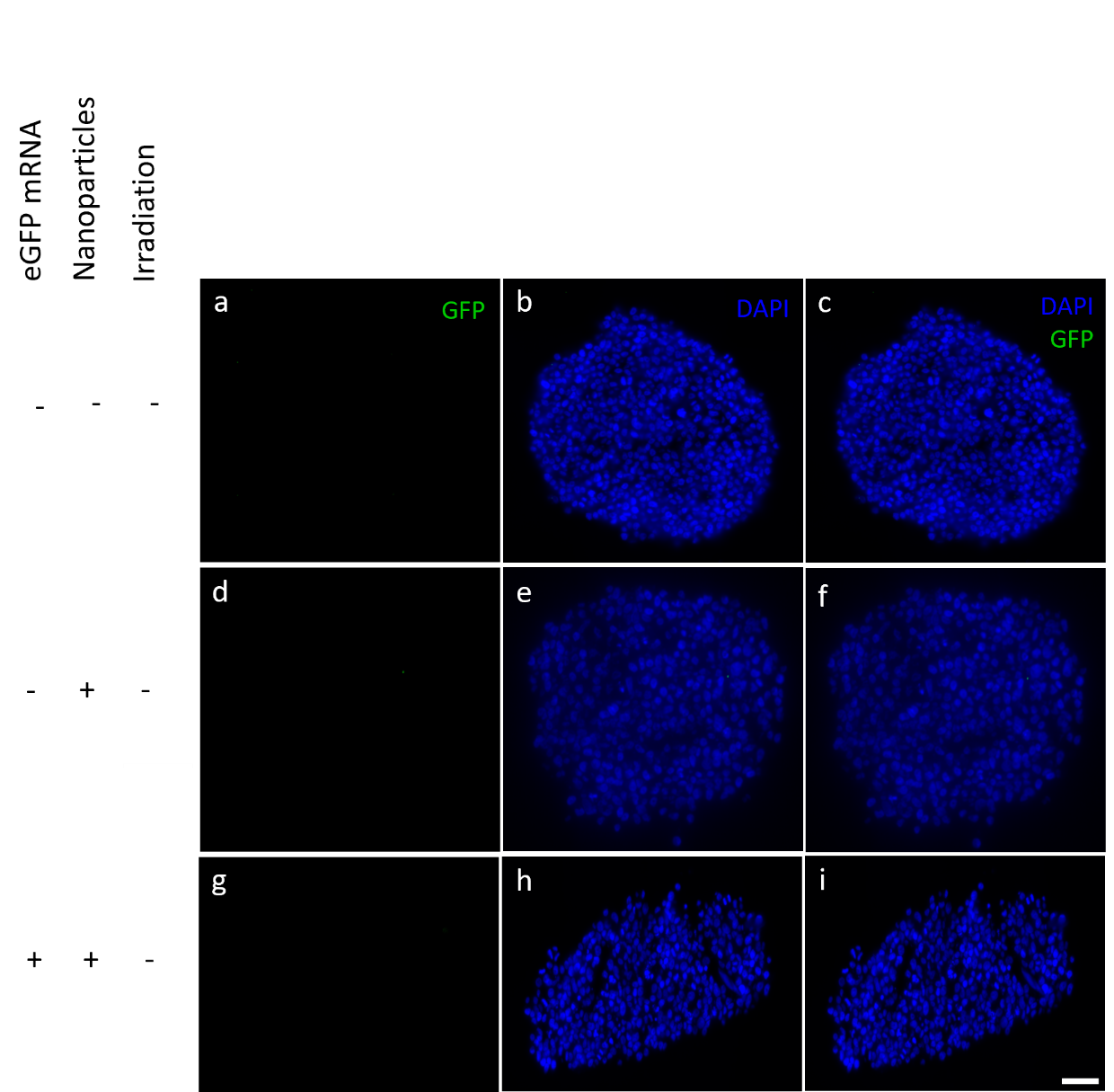


**Figure S8****: Controls for optoporation-mediated GFP mRNA delivery into MCF-7 spheroids. (a-c)** Spheroids without mRNA, nanoparticles or laser, **(d-f)** spheroids with nanoparticles but no mRNA or laser, **(g-i)** spheroids with mRNA and nanoparticles but no laser. Scale bar: 100 μm.
